# Supplementary figures and images for: Logistic PCA explains differences between genome-scale metabolic models in terms of metabolic pathways
Source: PLoS Comput Biol. 2024 Jun 24;20(6):e1012236. doi: 10.1371/journal.pcbi.1012236 (PMC11226097; doi:10.1371/journal.pcbi.1012236)

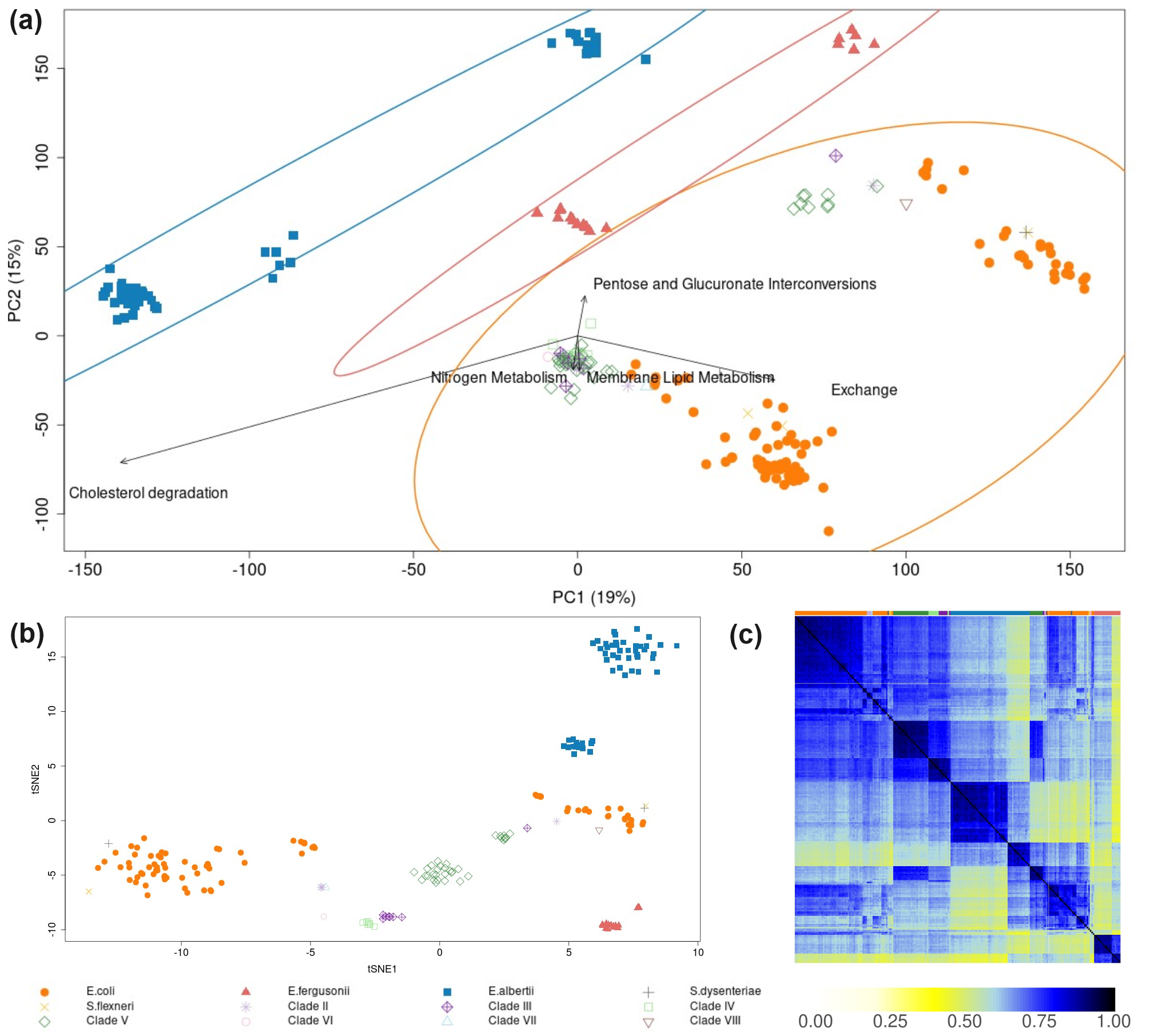

Supplement: S1 Fig — In panels (a) and (b), points represent individual GSMMs, with different genera indicated by distinct symbols and colors. The top row in panel (c) uses these same colors to indicate the corresponding genera. Circles in panel (a) highlight clusters of E. albertii strains (blue), E. fergusonii strains (red), and a mixed cluster of E. coli, S. dysenteriae, S. flexneri, and Clades II to VIII (orange). Labeled arrows in panel (a) denote subsystem-centric loading vectors from LPCA (refer to the results and methods section for definitions). The clustering of rows and columns in panel (c) was performed using the default hierarchical clustering settings (refer to methods section for details. (TIF) [file pcbi.1012236.s001.tif]

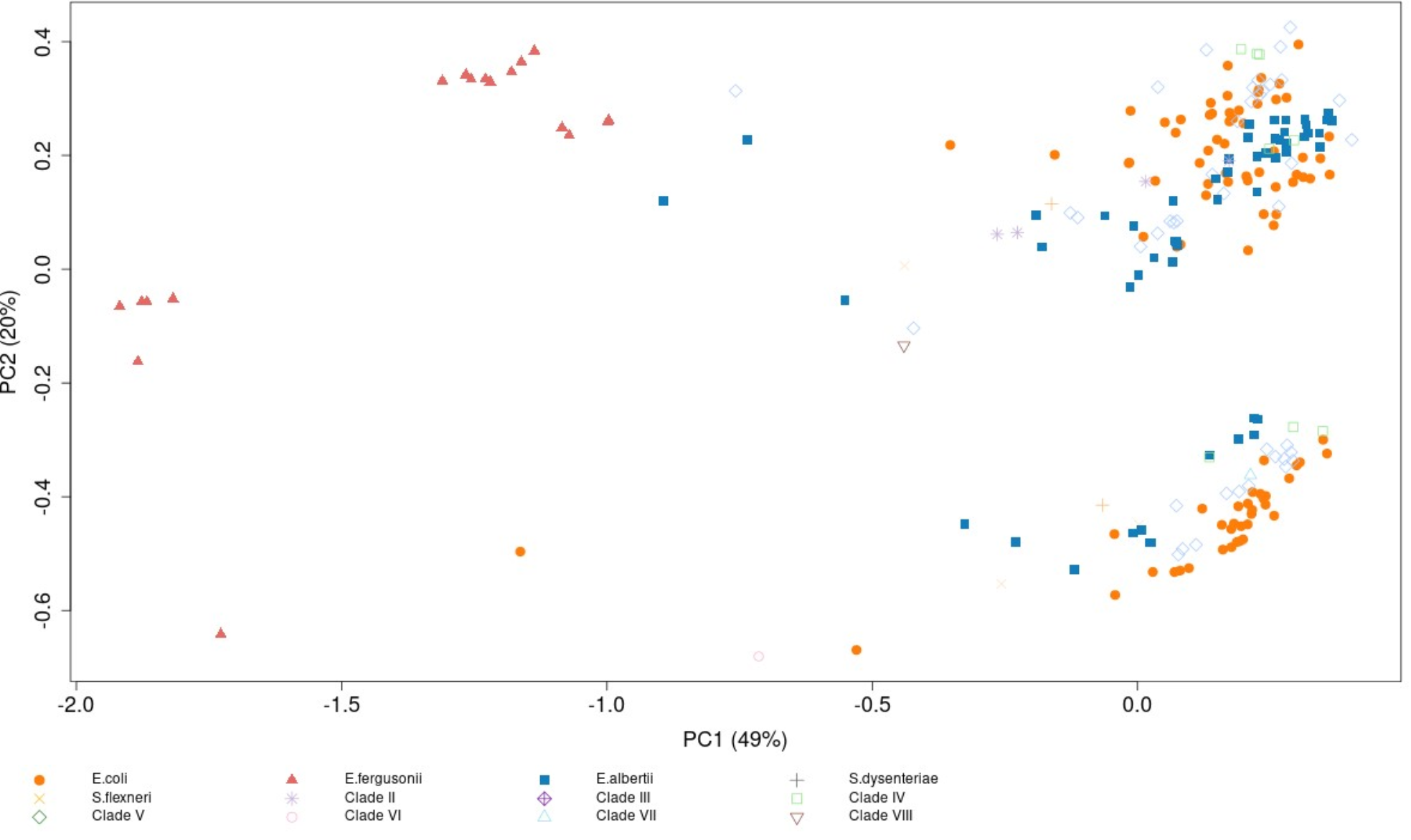

Supplement: S2 Fig — While E.fergusonii GSMMs could be well separated based on simulated growth rates, the remaining clades seem to be less separatable. (TIF) [file pcbi.1012236.s002.tif]

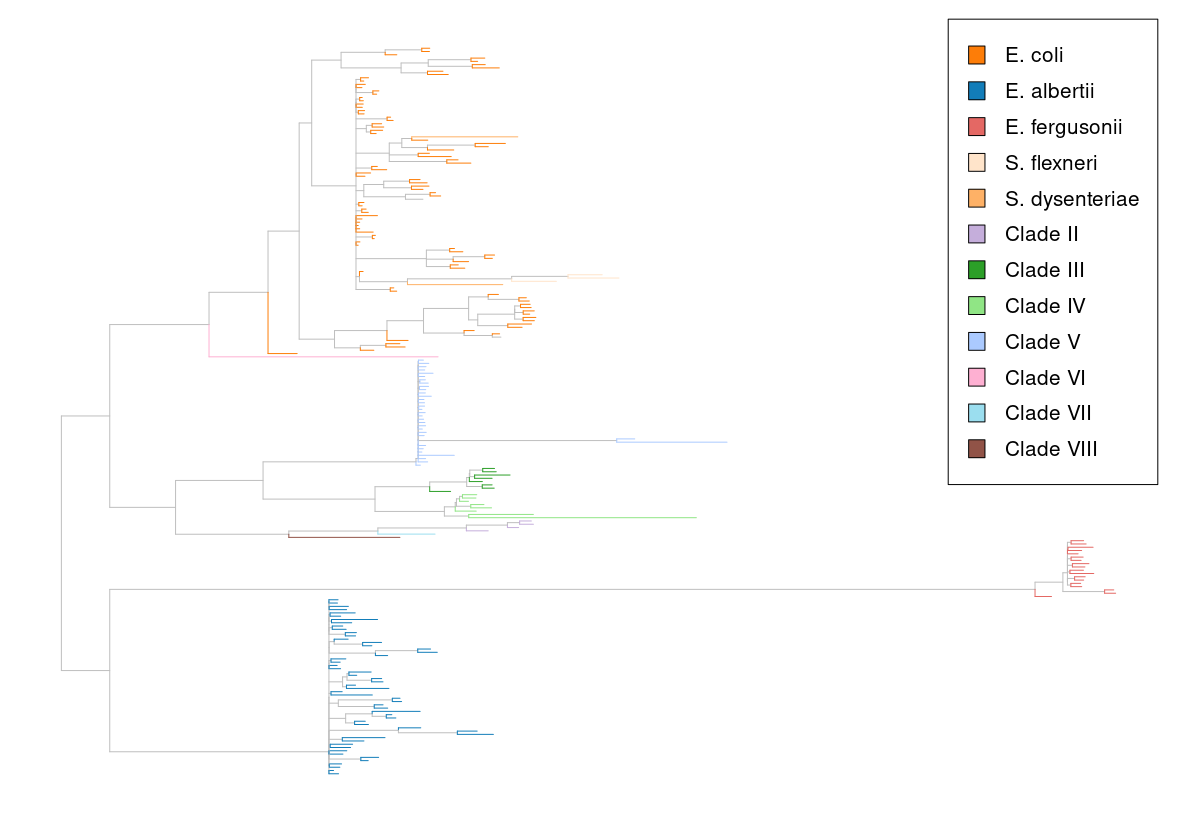

Supplement: S3 Fig — Genomes were obtained from Enterobase [30]. Phylogenetic relations were obtained from OrthoFinder [32] based on coding genes. E.coli, E.albertii, and E.fergusonii formed distinct clades. (TIF) [file pcbi.1012236.s003.tif]

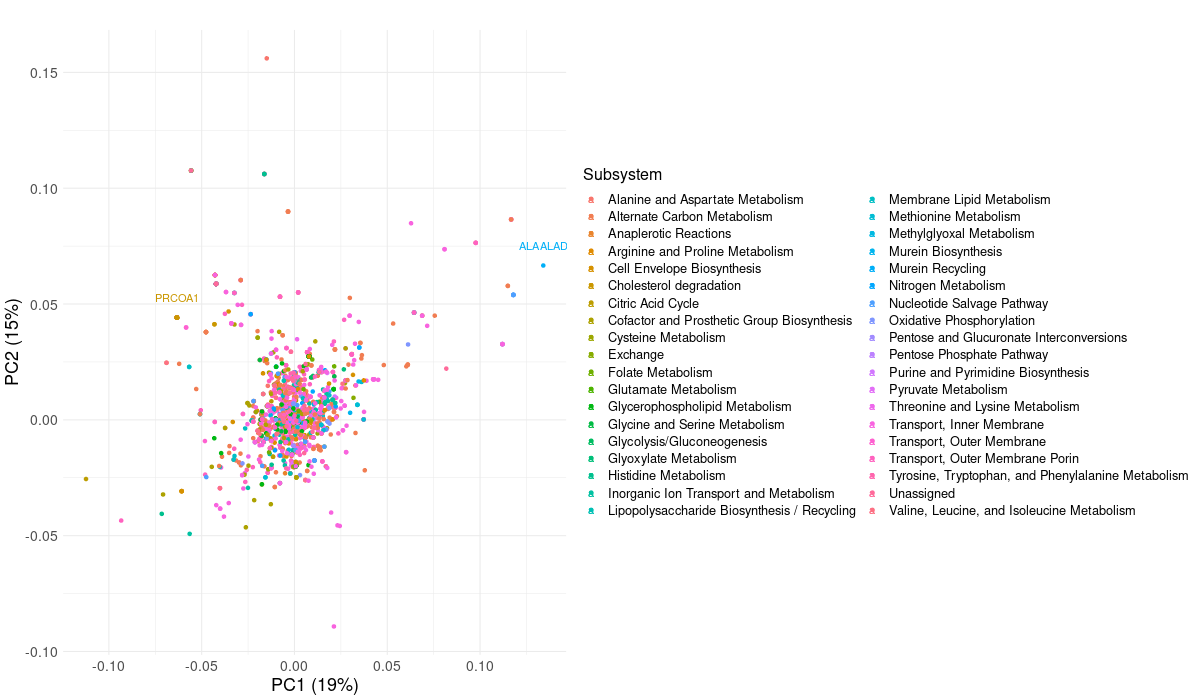

Supplement: S4 Fig — The reaction “ALAALAD” (Murein Biosynthesis) was found to be a major driving factor for separation. “PRCOA1” (Cholesterol degradation) was found to be incorrectly assigned to “Histidine metabolism” in the original GSMMs. (TIF) [file pcbi.1012236.s004.tif]

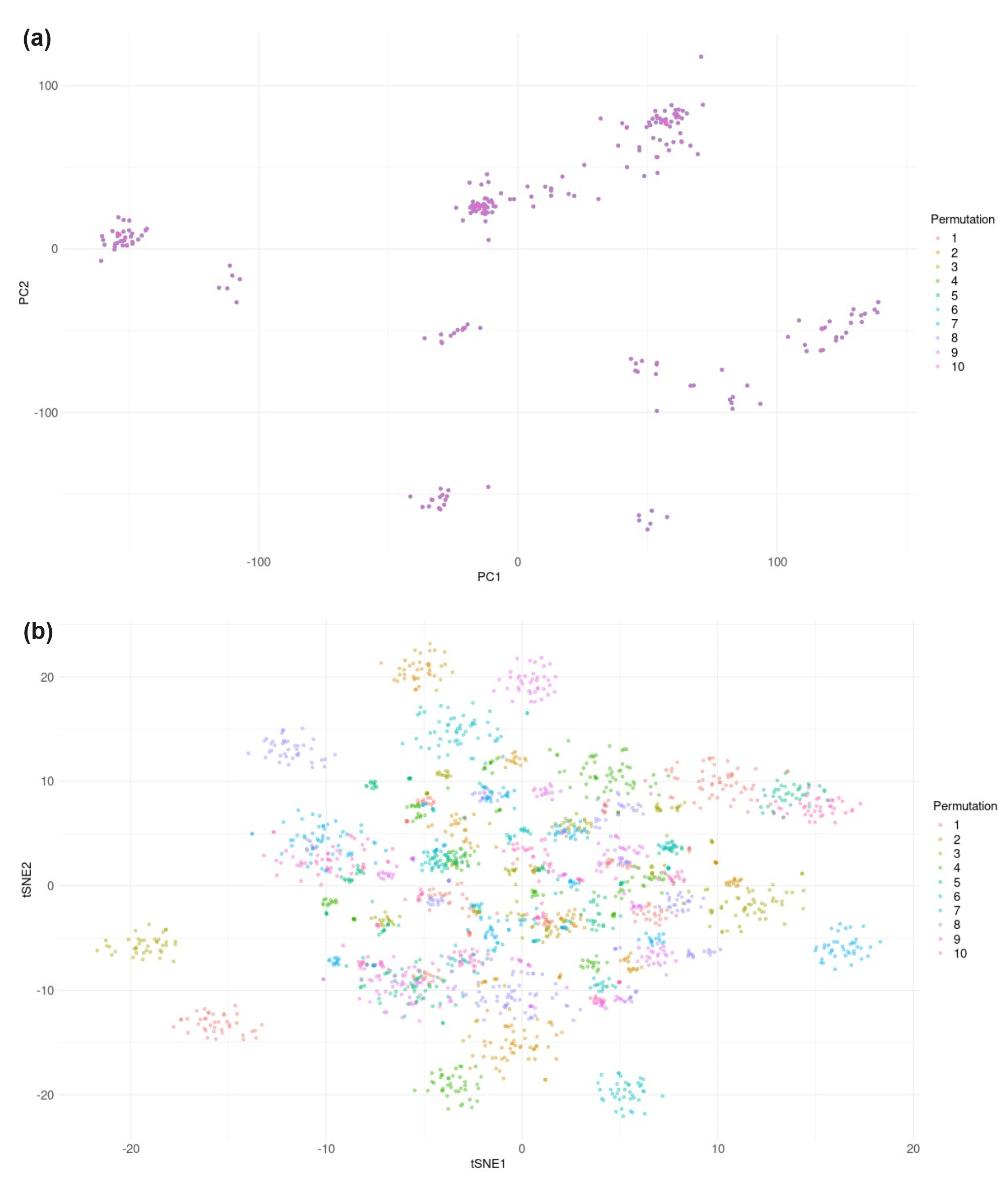

Supplement: S5 Fig — While LPCA scores showed reproducible clustering, t-SNE resulted in a more diffuse clustering. (TIF) [file pcbi.1012236.s005.tif]

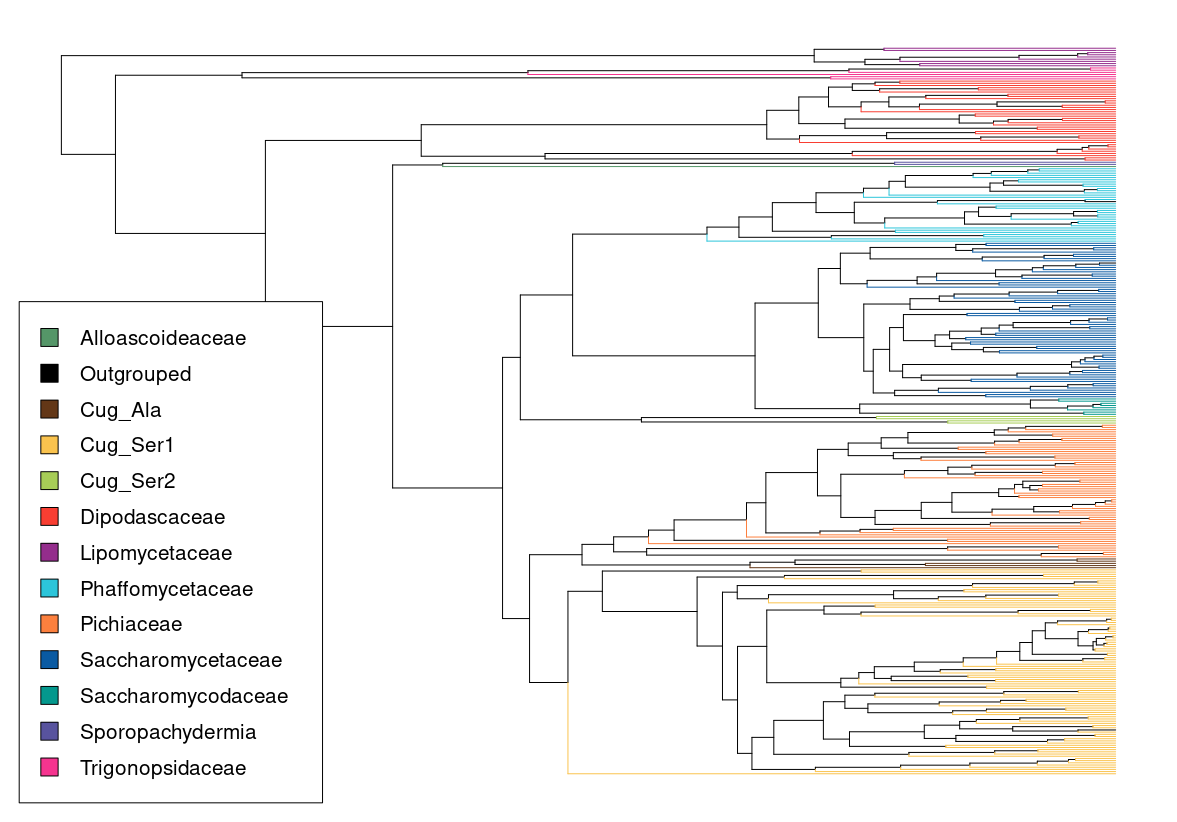

Supplement: S6 Fig — Whole-genome based phylogenetic comparison of strains results in more distinct separation of clades, compared to LPCA scores, t-SNE or hierarchical clustering based on Jaccard similarity. (TIF) [file pcbi.1012236.s006.tif]

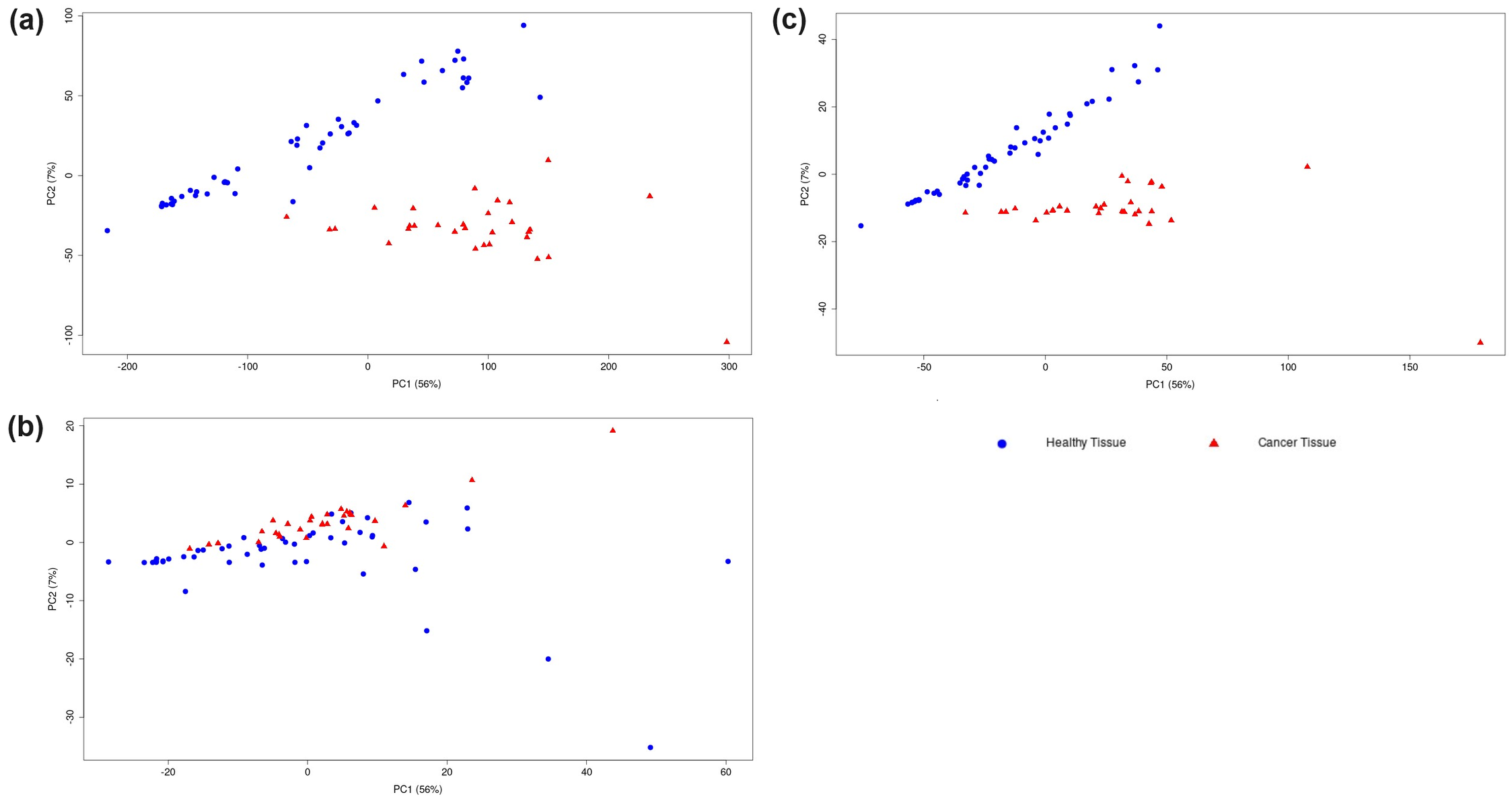

Supplement: S7 Fig — While clustering between healthy and cancer tissue could be conserved based on whole transcriptomes (a) and metabolic genes (b), it was not possible based on the genes from the context-specific reconstructions (c). (TIF) [file pcbi.1012236.s007.tif]
